# A Healthy School Start Plus for prevention of childhood overweight and obesity in disadvantaged areas through parental support in the school setting - study protocol for a parallel group cluster randomised trial

**DOI:** 10.1186/s12889-018-5354-4

**Published:** 2018-04-06

**Authors:** Liselotte Schäfer Elinder, Emma Patterson, Gisela Nyberg, Åsa Norman

**Affiliations:** 1 Department of Public Health Sciences, Karolinska Institutet, Tomtebodavägen 18A, 171 77 Stockholm, Sweden; 2 Centre for Epidemiology and Community Medicine, Solnavägen 1E, 113 65 Stockholm, Sweden

**Keywords:** Children, Diet, Low education, Parents, Physical activity, School health services, Type 2 diabetes

## Abstract

**Background:**

Systematic reviews conclude that interventions to prevent overweight and obesity in children obtain stronger effects when parents are involved. Parenting practices and parent-child interactions shape children’s health-related behaviours. The Healthy School Start Plus intervention aims to promote healthy dietary habits and physical activity and prevent obesity in children through parental support in disadvantaged areas with increased health needs, delivered by teachers and school nurses. This protocol describes the design, outcome and process evaluation of the study.

**Methods:**

Effectiveness of the intervention is compared to standard care within school health services. The 6-month programme, based on Social Cognitive Theory, consists of four components: 1) Health information to parents regarding the child; 2) Motivational Interviewing with the parents by the school nurse concerning the child; 3) classroom activities for the children by teachers; and 4) a web-based self-test of type-2 diabetes risk by parents. Effects will be studied in a cluster randomised trial including 17 schools and 352 six-year old children. The primary outcome is dietary intake of indicator foods, and secondary outcomes are physical activity, sedentary behaviour and BMI. Outcomes will be measured at baseline, at 6 months directly after the intervention, and at follow-up 18 months post baseline. Statistical analysis will be by mixed-effect regression analysis according to intention to treat and per protocol. Mediation analysis will be performed with parental self-efficacy and parenting practices. Quantitative and qualitative methods will be used to study implementation in terms of dose, fidelity, feasibility and acceptability. The hypothesis is that the programme will be more effective than standard care and feasible to perform in the school context.

**Discussion:**

The programme is in line with the cumulated evidence regarding the prevention of childhood obesity: That schools should be a focal point of prevention efforts, interventions should involve multiple components, and include the home environment. If effective, it will fill a knowledge gap concerning evidence-based health promotion practice within school health services to prevent obesity, and in the long term reduce social inequalities in health.

**Trial registration:**

The trial was retrospectively registered on January 4, 2018 and available online at ClinicalTrials.gov: No. NCT03390725.

## Background

Childhood obesity has been high on the public health agenda for two decades now and while the rapid increase in prevalence seen in the 1990s in the Nordic countries seems to have levelled off to about 3% in Sweden at the population level [1], huge social inequalities persist [2]. According to data from all 4-year-olds in Stockholm County born in 2012, collected through child health care services, the obesity prevalence varies from zero in the most well-off area to over 5% in the most disadvantaged [3]. The explanation for this gradient in younger children does not seem to be due to lower physical activity [4], but can to a large extent be explained by more unhealthy dietary habits in families with low levels of education [5] and of non-Nordic origin [6]. In adolescents, overweight and obesity often result in body image dissatisfaction, poorer self-assessed health status, social isolation, and decreased life satisfaction [7]. In addition, in high-income countries a negative association exists between educational attainment and obesity, particularly in women [8]. Although children in pre-school age [9] and early school-age [10] seem to be adequately physically active there is a clear decrease in physical activity with increasing age [11]. Evidence from Sweden [10, 12, 13] and elsewhere [14] suggest that children are less physically active in the home environment, e.g. during weekends.

When it comes to adults, higher rates of obesity and low physical activity are found in groups with low socio-economic position (SEP), and it has been speculated that these factors are the main causes behind the higher prevalence and earlier onset of type-2 diabetes (T2D) among adults with low SEP [15]. Furthermore, studies have shown that immigrants to Sweden have a significantly higher risk of T2D than Swedish-born individuals even when adjusted for established risk factors [16]. Today it is recognised that development of both obesity and T2D is driven by interacting genetic and environmental factors and is preventable to a large extent [17]. Because health-related behaviours and obesity often track from childhood to adulthood [18, 19], interventions should start from an early age and target the whole family.

The school environment in Sweden is relatively health promoting by international standards, with free school meals of good quality being served to all children [20], and spending time outdoors during the school day is generally encouraged. Therefore, the key to obesity prevention may be the home environment where parents shape the food, meal, and physical activity environment by being responsible for availability, timing, frequency of meals and activity, and through the way they interact with their children [21]. Indeed, several systematic reviews have concluded that interventions to prevent overweight and obesity in various settings obtain higher effects when parents are involved [22–27]. Research has identified different parenting practices and parent-child interactions which shape children’s health-related behaviours [28]. Practices like making food available in the home, serving as role models for healthy eating, and active and restrictive guidance show strong associations with healthy and unhealthy food consumption [29]. Regarding parenting practices to encourage children’s physical activity, logistic support and role modelling have been associated with higher activity in children [30]. In addition, our studies suggest that lack of parental cooperation and negative parent-child interactions may act as barriers to such healthy behaviours [31]. However, when it comes to social inequalities there are conflicting results regarding whether, and if so how parental feeding practices differ between different socioeconomic and cultural groups, and if these factors contribute to the social gradient in childhood obesity [32].

At the age of 6, almost all children in Sweden enter the school system to attend what is called the “pre-school class” and they have their first visit to the school health services together with their parents. Similar to the regular child health care visits during the preschool age (i.e. 0–6 years), this is an outstanding opportunity for health services to meet all families, regardless of SEP. It is a chance to boost parents’ knowledge and skills regarding health promoting practices at an important stage of the child’s life. National guidelines for school health services state that school nurses should actively work to promote health in cooperation with parents or guardians [33]. However, evidence-based programmes to promote health and prevent obesity within this setting are lacking.

Starting in 2010, our research group developed the Healthy School Start intervention, to be carried out in the school context, targeting parents and their 6-year-old children attending pre-school class. Two outcome studies have been published in geographical areas with mixed socioeconomic status [12] and in disadvantaged areas [10]. During this first school year the curriculum is flexible and the topic of diet, physical activity and health fit very well with the educational objectives. The Healthy School Start programme builds on Social Cognitive Theory (SCT) with parental self-efficacy and observational learning as central constructs [34]. The programme is in line with the latest evidence regarding the prevention of childhood obesity namely that schools should be a focal point of obesity prevention efforts, interventions should involve multiple components and include the home environment [26, 27]. The programme is complex and comprises several components such as health information to parents, motivational interviewing with parents and educational activities for the children followed by home assignments performed together with parents. The results of the first two trials were promising with regard to the children’s diet in comparison to standard care [10, 12]. In the second study, a transient decrease in BMI among children with obesity at baseline was also detected directly after the intervention ended [10]. However, most of the positive effects were not sustained at the 5-month follow up, indicating that the programme needs amplification to achieve sustained effects. The process evaluations showed that parents perceived the programme as compatible with family life and that teachers found the materials structured and easy-to-use, and not burdensome [35]. We learnt that in order to increase engagement among parents, the intervention components should be carefully tailored to the abilities of the participating families. Also, the importance of good cooperation between the home and the school, as well as within-family interplay became apparent.

We hypothesise that by enhancing the intervention, effectiveness will improve compared to our previous trials. In the new study we will increase the focus on parenting within each of the three components. Furthermore, we will add a new component addressing parents’ risk of developing T2D, which we believe will increase health awareness in families at higher risk. Furthermore, all intervention components will be carried out by school staff, which will improve the collaboration between the school and the home and facilitate future integration of the programme into routine practice. The objective of A Healthy School Start Plus study is to compare the effect of the programme to standard care on outcomes related to diet, physical activity and weight development of children. If successful, this programme will, in the long term, contribute to better health, lower social inequalities and societal costs for obesity and chronic diseases.

In this protocol we have followed the SPIRIT guidance [36] and for item 11 we used the TIDieR guideline [37].

## Methods

This intervention study will use quantitative and qualitative methods to evaluate both outcome and process and answer the following research questions:

1. What are the effects of the programme on children’s dietary habits, physical activity, sedentary behaviour and body weight compared to standard care?
2. How is the programme implemented with regard to dose, fidelity, feasibility and acceptability?
3. What are the mediators of the effects of the programme?

### Recruitment of schools, participants and setting

As a first step in the recruitment, key persons in municipalities in the Stockholm region were contacted, such as head school nurses, heads of elementary school, public health practitioners etc. They received a one-page invitation letter explaining the aim and content of the intervention and were invited to contact the project leader for more information. It was emphasised that the aim of the study was to improve health promotion practice in schools, rather than to burden them with extra work. In some cases the project leader was invited to the municipality or to a specific school to present the study to school staff. In other cases, the schools consented to participate and invited the project leader to the first ordinary school meeting with new parents, where parents are introduced to the school routines. Primary schools were eligible to participate if the proportion of parents with university education was less than 50% compared to 57% at national level [38]. Participating schools signed an agreement with the research team. In total 18 schools with 18 school nurses agreed to participate in the study. One school dropped out before recruitment of parents had started because the nurse went on sick-leave. Parents in the remaining 17 schools were subsequently recruited by the research team face-to-face or by telephone. All parents with a child in pre-school class were eligible to participate. Between May and September 2017, the project group was invited to the school’s meeting with new parents, who were informed about the intervention and the study. Written information was translated into the four languages most commonly used (Swedish, English, Somali and Arabic). Informed, written consent to participate in the study was obtained by the research team from all participating parents.

### Study design

The Healthy School Start Plus study will be conducted as a cluster-randomised parallel trial with randomisation at school level, with a wait-list control group. Simple randomisation of schools to the intervention or control group with a 1:1 allocation ratio was done using a computer-generated randomisation procedure by a statistician not involved in the study. As all school nurses in the intervention group had to undergo training in Motivational Interviewing (MI), something that takes a few months to reach a certain level of competence, randomisation took place before the baseline measurements in order to allow time for this training. However, allocation of schools to intervention or control was only revealed to headmasters, school nurses and the project leader, until baseline measurements done by research assistants, were finalised. Only then were teachers and parents informed. Baseline data was collected in September–October 2017 (T0) from 352 children. Data will again be collected 6 months after the baseline at the end of the intervention in April–May 2018 (T1) and at follow-up in April–May 2019 (18 months post baseline, T2). As an incentive to retain participants in the study, each family will receive a coupon for a healthy meal at a shopping centre restaurant. Mediators will also be measured on one occasion during the intervention, in February 2018 (TM). After the T2 measurement the control schools will be offered the intervention, including MI-training of the school nurses.

### The intervention

According to the typology of Benach et al. [39], the Healthy School Start Plus programme can be typed as a universal intervention with additional focus on the health gap. It will benefit all children in participating schools but due to the flexibility of the intervention components, extra attention can be given to families with higher needs. Based our previous experiences with the Healthy School Start programme, we added a fourth intervention component to the three existing ones. The four components are: 1) A health information brochure to parents; 2) one or two MI sessions with parents according to family needs and performed by the school nurse; 3) nine classroom activities performed by teachers with home assignments to be completed by children together with their parents; and 4) a web-based self-test of T2D risk for parents with feedback concerning the level of risk. Parents with an elevated risk will be encouraged via the website to visit primary health care for a health consultation according to existing procedures including testing, counselling and yearly follow-up, if diabetes or pre-diabetes is established. In this way, we hope to motivate high-risk families to improve health-related behaviours for the whole family.

Table 1 presents the programme theory of the Healthy School Start Plus study including intervention characteristics, process and outcome evaluation variables.

**Table 1:** Programme theory of A Healthy School Start Plus intervention study

Intervention characteristics				Process evaluation		Outcome evaluation	
Input	Core intervention components	Materials	Implementation strategies	Mediators	Process outcomes	Short term outcomes	Long term outcomes
Expert support Local capacity Funding	Health information MI with parents Classroom lectures and home assignments Parent’s test for T2D	Health brochure Teacher’s manual Child’s workbook	Written agreement with schools Kick-off meeting in schools Training of school nurses in MI Instructions for teachers in class room component	Parental self-efficacy Parental behavioural capability Parental outcome expectations and expectancies Child observational learning Motivation	Dose Fidelity to intervention components Feasibility Acceptability Context	Intake of vegetables and fruit Intake of unhealthy foods Physical activity Sedentary behaviour	BMI in children with overweight and obesity Waist circumference in children with overweight and obesity

The programme builds on SCT which explains behaviour as a reciprocal interaction between personal factors, the social, and the physical environment [34]. Several mediators affect behaviour change according to SCT, of which self-efficacy is a central one. Parental self-efficacy refers to parents’ beliefs in their capabilities to organise and execute specific tasks related to parenting of their child. Parental behavioural capability refers to a parent’s actual ability to perform behaviour through essential knowledge and skills. Parental outcome expectations refer to parents’ anticipated consequences of behaviour, whereas parental outcome expectancies focus on the value that parents place on the outcome. Observational learning occurs when children watch the actions and outcomes of role models’ behaviour like e.g. parents. Motivation is important to bring about change and is identified as a key process according to SCT in developing and executing agency in a person’s life, where self-efficacy and expectations play important roles.

The mechanistic links between the intervention, change in behaviour and health outcomes can also be described in terms of behaviour change techniques (BCT) [40]. According to a systematic review on the prevention and management of childhood obesity [41] it was concluded that six BCTs may be effective in obesity management interventions namely: 1) Provide information on the consequences of behaviour to the individual; 2) environmental restructuring; 3) prompt practice; 4) prompt identification as role model/position advocate; 5) stress management/emotional control training; and 6) general communication skills training. In prevention interventions prompting generalisation of target behaviour was shown to be effective. Since children in this study will be found in all weight status categories we expect that all seven BCTs will be relevant for our intervention. The BCT taxonomy [40] does not specify the interpersonal style of delivery, and MI is no longer included in the 2013 taxonomy. MI can be considered a combination of different BCTs of which several have been considered to be relational or content-based [42]. The hypothetical mechanistic links between each intervention component, the SCT-based mediators, and the relevant BCT are shown in Table 2.

**Table 2:** Description of hypothetical mechanisms of change by the intervention

Intervention components	Behaviour change techniques used^a^	Hypothetical mediators based on SCT	Parental practices targeted by intervention components^b^
Health information	4.1 Instruction on how to perform a behaviour 13.1 Identification of self as role model	Parental behavioural capability	Increase: Accessibility/facilitation/ healthy environment for healthy foods and physical activity Monitoring of behaviours Role modeling Involvement Encouragement in healthy behaviours Restriction of unhealthy foods and sedentary behaviours Co-participation in physical activity Decrease: Restriction of food and physical activity Pressure to eat or to perform physical activity Food for emotional regulation
MI with parents	38 relational and content-based BCTs [42] of which 16 overlap with Michie et al. [40] and 22 are original to MI In addition: 2.2 Feedback on behaviour 8.3 Habit formation 8.6 Generalisation of a target behaviour 11.2 Regulate negative emotions	Parental self-efficacy Parental behavioural capability Parental outcome expectations and expectancies Parental role modelling – observational learning Parent’s motivation	
Class room lectures and home assignments	8.1 Behavioural rehearsal/practice 13.1 Identification of self as role model (through home assignments)	Child’s motivation Parental self-efficacy Parental behavioural capability	
Parent’s test for T2D	1.4 Action planning 2.2 Feedback on behaviour 2.6 Biofeedback 5.1 Health consequences	Parent’s motivation	

^a^as decribed by Michie et al. [40] and Hardcastle et al. [42]

^b^as decribed by Masse et al. [65] Musher-Eizenman et al. [64]

### Intervention components

This complex intervention consists of four components which are described below. All materials are available on the project website (www.enfriskskolstartplus.se).

1. Health information: A 12-page brochure based on Swedish guidelines for diet and physical activity [43] contains facts and advice concerning parenting in relation to healthy food and family meal times, sweets, snacks, ice-cream, soft drinks, fruit and vegetables, physical activity, sedentary behaviour, screen time, sleep, and a theme regarding cooperation between parents. The text in the brochure was pilot-tested by parents and a final version was produced. The text is simple and short with many illustrations and available in Swedish, English, and Arabic. Parents will receive a printed version of the brochure from the school before the first MI session and it will also be available on the project website throughout the intervention.
2. MI is a client-centered, directive method for enhancing intrinsic motivation to change by exploring and resolving ambivalence [44] and is today regarded as an evidence-based counselling method for behaviour change in adults [45]. MI has previously been used in parental support interventions in the prevention and management of childhood obesity and to improve parenting skills [46–48]. The MI session will be scheduled by the school nurse as part of the ordinary health visit, where the child’s height and weight is measured, and which also includes a discussion about the child’s overall health. The aim of the MI session is to increase positive parenting and interplay regarding the child’s dietary and physical activity behaviour in the home environment. Parents in the intervention group will be offered one face-to-face MI session by the trained school nurse and another session, depending on need, as determined by the nurse. During the session (conducted without the presence of the child), the parents choose a specific behaviour that they wish to change or maintain. In line with MI, the parents are supported in identifying a target behaviour using an agenda-setting tool and in the exploration of personal values and the advantages of change or maintenance, and assisted in goal-setting if applicable. Each MI-session lasts for 20 to 30 min. School nurses will undergo MI training by trainers who are members of the Motivational Interviewing Network of Trainers (MINT). The training will be conducted prior to the start of the intervention and consists of 2 days covering MI theory and practical assignments, and two supervised recorded sessions, one of which is coded according the Motivational Interviewing Treatment Integrity Code protocol (MITI) [49]. In addition, the nurses will also receive supervision every other month on three occasions during the intervention. MI competence will be measured prior to start, post training and at the end of the intervention by using a standardised method where nurses perform MI via telephone with an actor, posing as a parent. Instruction materials and recordings with examples of MI sessions with parents will be available on the project website.
3. Classroom activities including home assignments. Nine manual-based classroom sessions of approximately 30 min duration will be delivered by the teacher. Various pedagogic materials are provided for the sessions including a workbook for children. Teachers will receive video-recorded instructions of how to use the teacher’s manual ahead of the start of the intervention. The homework aims to motivate the parents to exercise role modelling and supportive parenting practices related to diet and physical activity. The materials have been developed and pre-tested in collaboration with teachers.
4. The aim of the web-based T2D test is to make parents aware of their own risk of developing T2D. Parents complete a web-based test containing 8 questions. The test yields a score (the FINDRISC score [50]) between 0 and 26 points. Based on the result the parent is classified as being at low, somewhat elevated, medium, high or very high risk. In case of medium or higher risk (≥15 points) the parent will be advised by automatic feed-back to consult the local primary health care centre. The school nurse will not counsel the parent as this is outside of his or her remit. General information on health behaviours that minimise the risk for diabetes is provided to all parents who take the test, regardless of score.

In order to retain families in the study and to achieve a high level of adherence to the intervention components e-mails and text messages are sent to parents whenever the intervention enters a new stage, for example when the test for T2D becomes available on the website. Reassuring feedback regarding parental involvement in the intervention is included in each message sent by the research group.

### Control group

Children in the control schools will receive treatment as usual according to prevailing guidelines for school health services [33] and in addition parents receive the 12-page health information brochure (component 1 of the intervention). Usual treatment means a visit of the child accompanied by parents to the school nurse soon after the child begins school for a discussion regarding the school situation, the child’s overall well-being, relations with friends, possible health and learning problems, and diet and physical activity. The child’s height and weight is measured and eyesight and hearing are tested. If a child is overweight or obese, the guidelines for school health services state that the child should be met with an empathic approach with focus on improvement of health behaviours rather than on weight as such. No detailed instructions are given. The curriculum in pre-school class prescribes that pupils should be provided with opportunities to experience different forms of physical activity, and to develop an understanding of how health is affected and can be promoted in various ways [51].

### Outcome evaluation

All questionnaires are filled in via the project website (www.enfriskskolstartplus.se) with personal accounts for each family. The website has been developed by the research team in collaboration with IT consultants. One member of the research team is appointed administrator of the website. For parents who cannot or do not want to use the website, paper versions will also be available. In order to increase the response rate to the questionnaires, weekly reminders are sent out via e-mail and text messages. Parents are also offered support to log in to the website and if problems arise when responding to the questionnaires.

All outcomes are assessed as the difference between the intervention and the control group directly after the end of intervention at 6-months post baseline, and at follow-up 18 months post baseline, adjusted for baseline values. All outcome and mediator variables, indicators, instruments used and time points of assessment are listed in Table 3.

**Table 3:** Overview of outcome and mediator variables, indicators, instruments and time points of assessment

Variables	Indicators and instruments	Time point of assessment^a^
Demographic data parents	Sex, age, country of birth, years in Sweden, level of education, housing conditions, employment, weight, height, number of children	T0
Child’s diet	Child Eating Habits Questionnaire EQ [73] Revised version of Eating and Physical Activity Questionnaire (EPAQ) [53] Mobile phone based photo method based on Delisle et al. [54]	T0, T1, T2
Child’s physical activity and sedentary behaviour	Accelerometry Questionnaire: Active transport, activity in organisation, screen time on week days and weekends	T0, T1, T2
Child’s sleep	Revised version of Eating and Physical Activity Questionnaire (EPAQ) [53]	T0, T1, T2
Child’s BMI	Height and weight measured with instruments (SECA)	T0, T1, T2
Child’s waist circumference	Measured with instruments (SECA)	T0, T1, T2
Parental self-efficacy	Revised instrument based on Wright et al. [63]	T0, (TM), T1, T2
Parental feeding practices	Comprehensive Feeding Practices Questionnaire [64]	T0, (TM), T1, T2
Parenting practices regarding children’s physical activity	Items derived from item bank by Masse et al. [65]	T0, (TM), T1, T2
Nurses’ self-reported MI competence	Revised version of self-rating questionnaire based on MITI 4 [49]	TM
Parent-report of nurse’s MI competence	Revised version of self-rating questionnaire based on MITI 4 [49]	TM

^a^T0: baseline; TM: between T0 and T1; T1: 6 months after baseline; T2: follow-up 18 months after baseline

#### Primary outcomes

The primary outcome is intake at home of a composite score of indicator foods of importance for energy-balance and health, namely fruit and vegetables (healthy foods) and unhealthy foods (sweets, ice cream, buns/cakes, crisps) and unhealthy drinks high in added sugar (sugar-sweetened beverages). Intake of indicator foods was chosen as the primary outcome because our previous studies in comparable population samples showed that diet was a discriminating factor between different socioeconomic and ethnic groups [6] and also improved as a result of the intervention [10, 12]. Intake will be assessed using food frequency questionnaire, the Children’s Eating Habits Questionnaire (CEQ) which measures intake retrospectively in the home during the preceding month, and shows good reliability [52]. This food frequency instrument has been specifically designed for parents of children aged 2–11 years in eight European countries including Sweden and aims to investigate dietary behaviour and the family food environment. In addition, a revised version of the Eating and Physical Activity Questionnaire (EPAQ) will be used [53]. It measures the child’s intake of indicator foods in the home environment during the previous week, and is a semi-quantitative food frequency questionnaire, and includes portion size both in written text and in pictures. The original EPAQ measured intake during the previous day only and was validated against a 24-h recall in an Australian setting with parents of two to five-year-old children [53]. We will also use a new smartphone-based method to estimate the intake of indicator foods eaten at home (volume and frequency) prospectively through standardised photos taken by parents over two weekdays and one weekend day. This method constitutes a further development of the method developed by Delisle et al. [54]. Newer technology-based methods show promise for improving the accuracy of food records [55]. A relative validation study will be conducted in parallel during baseline, where all methods will be compared to multiple 24-h recalls conducted by independent dietitians as the reference method. Our intention is to use the photo-based method for the primary outcome in the intervention study if shown to be valid. The EPAQ will be used for comparison with our previous studies [10, 12].

#### Secondary outcomes

Secondary outcomes are physical activity and time spent sedentary, which will be measured objectively using accelerometry (GT3X+, Actigraph, LCC, Pensacola, USA) over 7 consecutive days which is considered valid and reliable [56]. The accelerometers are worn on a belt at the right hip and the children are instructed to wear the monitors during waking hours and to remove them for activities involving water. Children with at least 500 min of activity registration per day for a minimum of 3 days, including at least one weekend day, will be included in the analyses. Non-wear time will be defined as 60 min of consecutive zeros allowing for 2 min of non-zero interruptions. The epoch length will be set to 5 s. Cut-points for sedentary intensity will be defined as all activity below 100 cpm, moderate to vigorous intensity will be defined as all activity above 2296 cpm, and vigorous intensity will be defined as all activity above 4012 cpm [57]. A questionnaire with the following items will also be used: Is your child active in a club? How many times a week does your child participate in club activities? How does your child usually travel to school? How long does the travel take? How many hours per day does your child spend at the computer, tablet, smartphone (hours) during week days/weekends? Body composition (height, weight and waist circumference) will be measured using standardised procedures using SECA instruments by trained assistants, to a level of precision of 1 mm for height and waist circumference, and 100 g for weight. Waist circumference will be measured over the umbilicus, with the child standing upright with arms alongside the body and after exhalation. BMI status will be defined according to the International Obesity Task Force [58]. BMI standard deviation score (BMI sds) will be calculated according to a Swedish reference standard [59] and according to International Obesity Task Force references [58]. One item from the EPAQ will be used to estimate sleeping hours in the children by parental report [53].

#### Covariates

Parental educational level and occupation is self-reported. Based on previous experience, we anticipate that a high proportion of parents will have a non-Swedish background and therefore a composite measurement of SEP is likely to be more appropriate. The composite measurement will consist of the highest level of education attained, and the occupation (according to Statistics Sweden) by either of the parents. A dichotomised variable indicating low and high SEP will be created. Parents will also be asked to indicate their country of birth as well as number of years living in Sweden. Country of birth will be classified as “Sweden/the Nordic region”, “Europe” or “outside Europe”. In addition, child sex will be used.

### Process evaluation

The process evaluation is based on the MRC guidance by Moore et al. [60]. The process evaluation will include intervention dose, fidelity, feasibility, acceptability and a description of the context.

#### Dose

The dose will be estimated quantitatively in the following way. Parents will be asked in a non-judgmental way by the school nurse if they have read the brochure. The number of MI sessions per family will be noted. Compliance with the teaching sessions and workbook completion will be monitored by teachers in a logbook, documenting core elements of the lesson, how much time they spent on each lesson, and whether they made any adaptations. The result of the T2D test will be available from the website.

#### Fidelity

Fidelity to the MI component will be assessed as follows. All MI sessions will be recorded and a random selection of 10–20% will then be coded for MI competence using the MITI protocol [49] by reliable coders in a coding lab at Karolinska Institutet (MIQA group). The MI competence of all nurses in the intervention and control group will be assessed before the first MI training in May 2017 by recording them having a standardised conversation with a trained actor posing as a parent. This will be repeated after MI training in August/September 2017, and again after the end of intervention in May 2018.

The school nurses in the intervention group will report the self-perceived MI quality of each MI session with parents. After each session the nurses will appraise their level of empathy they projected during the session, how much they focused on evoking change talk from the parents, and how much they used ‘reflections’ as opposed to posing questions. Parents will respond to an equivalent questionnaire after the MI session where they rate their perception of the school nurse’s expression of empathy and ability to motivate the parent to improve practices.

#### Feasibility and acceptability

After the end of the intervention, semi-structured interviews with parents, school nurses and head teachers will be performed to explore their views on the acceptability and feasibility of the intervention. In addition, focus groups will be conducted with the children to explore their perceptions of the activities in class and the home assignments. As an incentive to participate in the focus groups, children will receive a gift card to a book shop worth about 10 euro. Qualitative methods permit the researcher to study selected issues in depth and detail [61]. The interview guides will be based on the five interrelated domains of implementation (intervention characteristics, outer setting, inner setting, characteristics of the individuals involved, and the process of implementation) of the Consolidated Framework for Implementation Research (CFIR) [62]. This framework provides a pragmatic structure for analysing implementation factors in complex, multi-level interventions. Based on the findings, implementation strategies will be adjusted, if necessary, and a final manual will be produced after the end of the study.

#### Mediators

The potential mediators parental self-efficacy and observational learning/role modelling will be measured by questionnaires in both mothers and fathers. A revised and culturally adapted version of the 16-item questionnaire assessing parental self-efficacy regarding children’s diet and physical activity [63] will be used. The original instrument was validated in a sample of individuals mainly with low SEP and has been adapted by our research group to parents with a heterogeneous cultural background by changing the content and wording of some items. The instrument assesses parental self-efficacy for influencing physical activity during the weekend, intake of vegetables, sweets and chocolates, and sugar-sweetened beverages, each in three challenging situations. Parental feeding practices, including modelling, will be measured using the Comprehensive Feeding Practices Questionnaire (CFPQ) covering both positive and negative practices and validated in a middle-income US sample [64]. The questionnaire will assess the parental feeding practices of involvement, environment, food restriction for weight control, restriction for health reasons, encouragement of balance, pressure to eat, monitoring, emotion regulation, food as reward, and modelling. Few instruments exist to assess parenting practices regarding children’s physical activity and none of the existing ones show acceptable psychometric properties. Therefore, 25 items will be used from an item bank currently being developed for this purpose [65]. The items cover the parenting practices modelling, pressure, encouragement, guided choice, involvement/praise, co-participation, facilitation, monitoring, and restriction. All questionnaires have been translated into Swedish and back-translated by the research team and pre-tested in parents with pre-school children.

### Data management

All participants will receive a code and data will be depersonalised. The linking code will be kept separate from the data in a secure and locked place. Data collected during anthropometric measurements will first be written on paper and subsequently entered into the database by the research group. Original paper documentation will be saved and stored securely at Karolinska Institutet to allow for quality control of electronical data entry. Data collected though photos will be coded according to a standaridised operating procedure by dieticians/nutritionists and tested for inter-rater reliability on a regular basis. Data gathered using accelerometry will be analysed with the ActiLife software programme. Original data will be stored electronically.

All data collected through the project website and otherwise will be stored electronically in password protected folders on a secure data server at Karolinska Institutet to avoid unauthorized access. Folders containing raw data will be stored separately from the data being used in analysis. Measures to prevent loss of data will be taken through the use of systematic back-up routines throughout data collection and by data storage on servers with complete data back-up on a daily basis. Access to data will be restricted to the research personnel working directly with data entry or analyses.

### Data analysis

A detailed statistical analysis plan will be developed in collaboration with an academic statistician prior to the start of the data analysis. Here we describe the key elements. Differences in continuous demographic variables at baseline between intervention and control group will be presented as means ± SD and tested using independent t-tests for normally distributed data. Non-normally distributed variables will be presented as median and range and will be analysed by non-parametric tests. For categorical variables, proportions will be presented and differences will be analysed by chi-square test. Effectiveness with regard to primary and secondary outcomes will be analysed by mixed-effect regression analyses with two levels (individual and school) with data at T1 or T2 as outcomes, according to the principle of intention to treat. We will first test a crude model for all outcomes at T1 or T2 with group as the predictor and adjusted for baseline values of the relevant outcome. In a second step, sex and parental SEP will be added to the model. Interaction between group and sex or group and parental SEP will be tested and analyses stratified if significant interaction terms are found. All children with measurements at T0 will be included and missing data will be handled with multiple imputation. The effect that any missing data might have on results will be assessed by performing sensitivity analyses of augmented data sets. Analysis will also be performed per protocol including only those children whose parents have attended at least one MI session.

Subgroup analyses will be performed with regard to baseline BMI status (underweight plus normal weight, overweight, obesity) and waist circumference (dichotomised). In addition quantile regression will be performed to detect differential intervention effects according to baseline BMI quartiles. The hypothesis behind the subgroup analysis is that children with a high BMI at baseline will respond with a decrease in BMI in contrast to children with lower BMI. Regarding physical activity (TPA, MVPA and time spent sedentary) outcomes will be adjusted for accelerometer monitor wear time.

Mediation analysis will be performed according to MacKinnon [66] with parental self-efficacy and parenting practices, including modelling, as the hypothetical mediators, to see if the programme works according to its theory, and whether effects are moderated by child sex or SEP. All statistical analysis will be performed blinded to group allocation.

Interviews and focus groups will be transcribed verbatim and analysed using qualitative content analysis [67].

### Sample size calculation

Dietary intake of unhealthy foods is the primary outcome. In our previous study [10], the intake of unhealthy foods was 1.7 ± 2.0 portions/day. We now aim for a difference in intake of unhealthy foods of 0.8 portions/day between the intervention and the control group. Sample size calculation was done according to Twisk [68]. With a power of 80% and significance level of 5% and an intra-class correlation coefficient of 2% (clustering at school level), we need to recruit at least 17 schools with 15 children in each school, 255 children in total.

### Ethics approval and consent to participate

The study will be conducted according to the guidelines laid down in the Helsinki Declaration and was approved by the Regional Ethical Review Board in Stockholm No. 2017/711–31/1. Consent to participate in the study has been obtained from all parents, nurses and teachers. This intervention is not expected to have any adverse effects.

## Discussion

This multi-disciplinary theory-based programme will fill a large knowledge gap regarding evidence-based practice within school health services to promote health-related behaviours and prevent overweight and obesity in children. The programme has been designed to be fully integrated into the usual school routines, making it highly sustainable once the necessary support from the school management can be secured. Taking a universal approach yet tailored to the special needs of families, the programme will have high external validity and with the potential to reduce social inequalities in health. The project will also for the first time establish a link between the school health services and primary health care, thus reaching the whole family. Measures to prevent obesity and T2D are especially important in areas with low SEP where obesity is several-fold higher, and where T2D starts 6–8 years earlier among adults than in high-income areas [15].

One of the study’s strengths is the well-developed programme theory and the description of the hypothetical mechanism of change as well as the strong study design. Furthermore, MI fidelity will be monitored and reported. A number of studies have used MI to target parents regarding children’s diet and physical activity and prevention or treatment of overweight and obesity [69–72] with mixed success. However, none of these reported fidelity to MI. In the present study we will train school nurses well ahead of the intervention and follow their learning process. In additional studies we will relate their MI competence to the effectiveness of the intervention.

The main weakness of the study is that the primary outcome (dietary intake of indicator foods in the home environment) is based on self-report as are the theory-based mediators. Assessing diet, especially in children, remains extremely challenging due to lack of feasible objective methods. We intend to use and validate a photo-based method to estimate selected food items and quantities within this study. We hope that this method will be acceptable to the target group of culturally diverse families many of whom have a low level of education. As a backup method we will use the Child Eating Habits Questionnaire EQ [73] developed collaboratively in eight European countries.

Another weakness is that we had to randomise schools to intervention and control groups before the baseline measurement was performed. This was necessary as nurses in intervention schools had to undergo a training period and because the intervention had to follow the school year. However, we did our best to keep intervention allocation hidden from other school staff and research assistants until baseline measurements were finalised. For obvious reasons, blinding of participants, nurses, teachers and the research team was not possible after the intervention had started which could lead to biased responses and differential response rates among participants. However the data analysis will be performed blinded to group allocation.

Mixed-methods will be used in the study to evaluate the implementation process. Qualitative methods permit the researcher to study selected issues in depth and detail, such as e.g. positive and negative views held by participants regarding the intervention and possible unwanted effects. Mixed methods are recommended when conducting pragmatic (real-world) trials, because they can reveal if a potential lack of effect is caused by intervention failure or implementation failure or both. In agreement with the UN Convention on the Rights of the Child we will interview the children about their experience and perception of this intervention.

## Conclusion

While obesity and chronic diseases can be managed or treated in many cases, early prevention remains the most sustainable option by far from both an ethical and an economic perspective and more effective methods of prevention have repeatedly been called for by the World Health Organization [19] and many others [18]. If effective, wide-scale implementation of this programme in schools according to the principle of proportionate universalism [39], with actions at scale but at intensity proportionate to needs, could lead to better health and lower health inequalities due to unhealthy diets and low physical activity in the population.

## Abbreviations

BCT: Behaviour change techniques
MI: Motivational Interviewing
MITI: Motivational Interviewing Treatment Integrity code
SCT: Social cognitive theory
SEP: Socioeconomic position
T2D: Type-2 diabetes
